# *In vitro* digestions and IgE binding of proteins from white and whole hen’s egg

**DOI:** 10.1186/2045-7022-1-S1-O8

**Published:** 2011-08-12

**Authors:** Gustavo Martos, Rosina López-Fandiño, Elena Molina

**Affiliations:** 1 Institute of Food Science Research (CIAL, CSIC-UAM), Madrid, Spain

## 

Egg is leading the top 8 allergenic foods in infants. During gastrointestinal digestion, various factors can affect the proteolysis of food allergens, such as interaction with lipids or bile salts. Therefore, the amount of immunologically active protein reaching the intestinal mucosa can be substantially modified. Here we investigated egg white digestion under physiological conditions, including presence of bile salts and phosphatydilcholine. We compared it to whole egg digestion and sought resistant proteins or fragments that react with IgE antibodies in allergic children's sera.

Whole hen's egg and egg white were subjected to an in vitro gastroduodenal digestion consisting on a gastric pepsinolysis at pH 2, followed by a duodenal proteolysis at pH 7 with trypsin and chymotrypsin. Digestion patterns were analysed by RP-HPLC and 1D/2D SDS-PAGE . The binding of the digests to IgE present in a pooled serum from egg allergic children was investigated by western blotting.

Among the major egg white allergens, ovalbumin (OVA) and lysozyme (LZ) were the most resistant to gastroduodenal digestion, whereas ovotransferrin (OVT) and ovomucoid (OM) were rapidly hydrolyzed during pepsinolysis. Three peptides were found to be the most resistant in the presence of phosphatidylcholine during duodenal digestion. Regarding the whole egg, the most abundant egg yolk protein, lipovitellin I, was partially hydrolyzed by pepsin and completely digested by duodenal enzymes in the presence of physiological amounts of bile salts. Coupling 2D electrophoresis and immunoblotting, we detected five major proteins highly immunoreactive. Besides the already known allergens OVA, OM, OVT and LZ, we also found ovoinhibitor strongly reacting with serum IgE. This minor protein has rarely been referred as an allergen. After 60 minutes of egg white duodenal digestion, there remained several resistant peptides of intermediate molecular weight (10-40 kDa) capable of binding IgE.

